# Tissue levels of active matrix metalloproteinase-2 and -9 in colorectal cancer

**DOI:** 10.1038/sj.bjc.6600366

**Published:** 2002-06-17

**Authors:** E T Waas, R M L M Lomme, J DeGroot, Th Wobbes, T Hendriks

**Affiliations:** Department of Surgery, University Medical Centre St Radboud, Nijmegen PO Box 9101, 6500 HB, The Netherlands; Gaubius Laboratory, TNO Prevention and Health, Leiden PO Box 2215, 2301 CE, The Netherlands

**Keywords:** matrix metalloproteinase, colorectal cancer, gelatin zymography, gelatinase activity assay

## Abstract

The bioactivity of matrix metalloproteinases was studied in tissues from colorectal cancer patients by means of both quantitative gelatin zymography and a fluorometric activity assay. Next to paired samples of tumour tissue and distant normal mucosa (*n*=73), transitional tissue was analysed from a limited (*n*=33) number of patients. Broad-spectrum matrix metalloproteinase activity and both the active and latent forms of the gelatinases matrix metalloproteinase-2 and -9 were higher in tumour than in normal mucosa. The ratio's between active and latent forms of matrix metalloproteinase-2 and -9 were highest in tumour tissue and normal mucosa, respectively. Matrix metalloproteinase-2 levels, both active and latent forms, correlated inversely with stage of disease, the tumours without synchronous distant metastases containing significantly (*P*=0.005) more active matrix metalloproteinase-2 than the others. At much lower levels of activity, the same trend was observed in distant normal mucosa. The level of latent form of matrix metalloproteinase-9 in tumour depended on tumour location. Neither the active form of matrix metalloproteinase-9 nor broad-spectrum matrix metalloproteinase activity in tumour tissue did correlate with any of the clinicopathological parameters investigated. The results demonstrate explicit differences between the activity of matrix metalloproteinase-2 and -9, indicating different roles for both gelatinases in tumour progression. Such data are necessary in order to develop rational anti-cancer therapies based on inhibition of specific matrix metalloproteinases.

*British Journal of Cancer* (2002) **86**, 1876–1883. doi:10.1038/sj.bjc.6600366 www.bjcancer.com

© 2002 Cancer Research UK

## 

Proteinase activity is an important feature in a multitude of physiological and pathological processes. Proteinases are normally strictly regulated in order to prevent undesired degradation of tissue components. In pathological processes like arthritis, periodontal disease, liver fibrosis, cardiovascular disease and cancer, the regulation of proteinase activity is disturbed (Nagase and Woessner, 1999). In cancer, degradation of the extracellular matrix is a key event in tumour cell invasion and metastasis. Various members of the matrix metalloproteinase (MMP) family have been shown to play an important role in these processes, for instance by facilitating the entry of tumour cells into the bloodstream, angiogenesis, tumour cell establishment and growth (Curran and Murray, 2000; Nelson *et al*, 2000). A good understanding of the expression pattern of the various MMPs in specific types of cancer may provide a basis for the development of new therapeutic strategies. For this purpose, data on the activities of individual MMPs in tumour tissue are needed.

In a number of studies a positive correlation has been demonstrated between MMP expression and tumour invasion and metastasis. Data pertain to different types of cancer located in various organs (Curran and Murray, 2000). The assay methods used vary widely and include analysis of mRNA, protein content and actual proteinase activity. In colorectal cancer (CRC) most of the data are on mRNA, which is upregulated in tumour cells and/or tumour stroma, if compared to normal healthy tissue. For instance, upregulation of mRNA has been reported for MMP-2 (Pyke *et al*, 1993), MMP-7 (Mori *et al*, 1995), MMP-9 (Zeng *et al*, 1996), MMP-11 (Thewes *et al*, 1996), MMP-12 (Yang *et al*, 2001) and MMP-14 (Sardinha *et al*, 2000). In the immunohistochemical detection of MMPs the focus has been on the analysis of MMP localisation. Correlations have been described between MMP-1 immunoreactivity and survival (Murray *et al*, 1996) and also between stage of disease and immunoreactivity for MMP-1 (Shiozawa *et al*, 2000), MMP-2 (Levy *et al*, 1991) and MMP-7 (Adachi *et al*, 1999). These studies measure MMP immunoreactivity without discrimination between latent proenzyme and activated enzyme. Ultimately, quantitative data on proteinase activity are most relevant for determining the biological role of MMPs in tumour progression.

Quantitative gelatin zymography allows the simultaneous measurement of the latent and active forms of the gelatinases MMP-2 and MMP-9. In recent years, a limited number of studies used zymography to investigate the presence of the gelatinases in tissue samples collected from patients operated for CRC. To some degree, all show enhanced gelatinase levels in tumour tissue, at least for the latent forms of MMP-2 and MMP-9. However, some studies (Liabakk *et al*, 1996; Parsons *et al*, 1998) have not quantified active MMP-9, while others (Garbett *et al*, 1999a; Zeng *et al*, 1999; Baker *et al*, 2000) do not report specific activities but rather the absence or presence of the various enzyme forms. A consistent quantification of both the latent and active forms – and their ratio – of both gelatinases in matched tumour and normal tissue is still lacking. Here, we supply these data on samples from a series of CRC patients, which is also large enough to investigate correlations with clinicopathological parameters. In a number of cases we have also measured activities in transitional tissue containing the invasive edge. In addition to quantitative gelatin zymography, we have used a recently developed broad-spectrum bioassay MMP activity using an internally quenched fluorogenic peptide substrate.

## MATERIALS AND METHODS

### Patients and tissue samples

Seventy-three patients with CRC were included in this study. The paired samples of tumour tissue (*n*=73), transitional tissue (*n*=33) and distant normal mucosa (*n*=73) were collected immediately after surgical resection for primary CRC. Macroscopically vital tumour tissue from the protruding luminal part of the tumour was harvested by a pathologist. Transitional tissue contained the invasive edge on the luminal side including both tumour and normal tissue. Normal colonic mucosa samples were taken at least 5 cm away from the tumour.

Randomly selected samples were analysed histologically to confirm their cellular content. Samples of patients that received pre-operative chemo- or radiotherapy were excluded from the study. All patients provided informed consent and the study was approved by the institutional ethical committee. The patients' clinicopathological characteristics were determined and used to compose various groups for comparison (c.f. Table 2

**Table 2:**
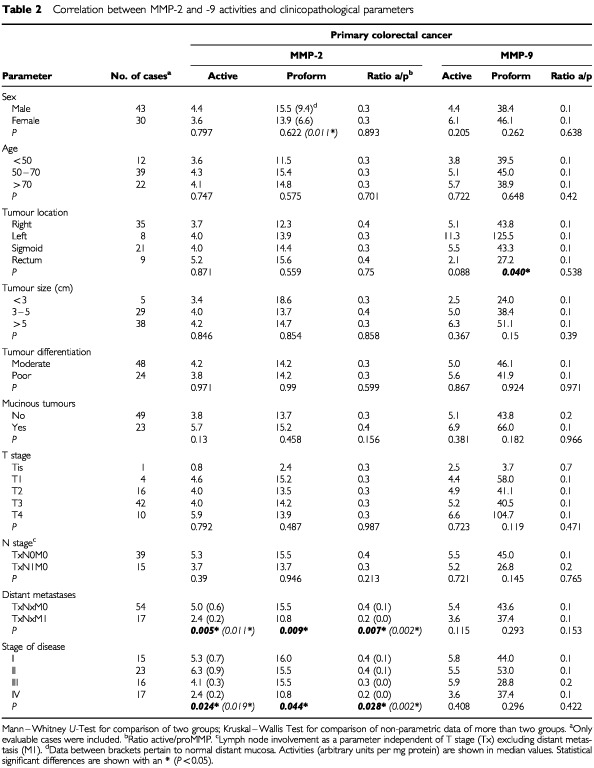
Correlation between MMP-2 and -9 activities and clinicopathological parameters

). Tumours were designated mucinous when significant parts of the tumour contained clusters of enlarged mucin producing cells. Tumour staging was according to the current TNM classification, stage I (T1-2N0M0), II (T3-4N0M0), III (TxN1-3M0) and IV (TxNxM1) corresponding to Dukes' stage A, B, C and D, respectively.

Tissue samples were stored in liquid nitrogen and mechanically disaggregated using a Micro-Dismembrator (Braun), yielding homogenous samples. The pulverised samples were extracted (40 μl per mg tissue) in buffer (0.5 M Tris, 0.2 M NaCl, 10 mM CaCl_2_ and 1% Triton X-100) for 15 min and freeze–thawed twice. After centrifugation (18 000 r.p.m., 30 min, 4°C) the supernatant was dialysed twice (20 h, 4°C) against a buffer containing 5 mM CaCl_2_, 50 mM Tris-HCl and 0.2 M NaCl to remove Triton and the excess of salt. Tissue extracts were stored at −80°C until use.

### Protein determination

Protein content was determined in tissue extracts using the bicinchoninic protein assay from Pierce (Rockford, IL, USA). To each extract of 20 μl, 130 μl buffer (50 mM Tris, 0.2 mM NaCl, 5 mM CaCl_2_, pH 7.5) and 2 ml of bicinchoninic acid solution was added. Following incubation for 30 min at 37°C the absorbance was read at 550 nm. Bovine serum albumin was used as a standard.

### Gelatin zymography

**Figure 1:**
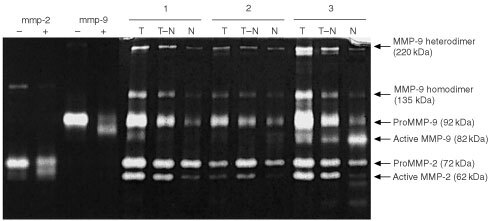
Proteolytic MMP activity in human CRC tissue extracts detected by quantitative gelatin zymography. MMP-2 (−), gelatinolytic activity of recombinant proMMP-2; MMP-2 (+), APMA-activated recMMP-2; MMP-9 (−), recombinant proMMP-9; MMP-9 (+), APMA activated recMMP-9; T, tumour; T–N, transitional (tumour to normal) tissue; N, distant normal mucosa; 1–3, case numbers of three representative patients. Positions of active MMP-2 (62 kDa), proMMP-2 (72 kDa), active MMP-9 (82 kDa) and proMMP-9 (92 kDa) are indicated by arrows. Recombinant MMP-2 and -9 were applied in doses of 1 and 0.5 ng per lane, respectively.

Gelatin zymography was performed to quantify the presence of both activated and latent forms of the gelatinases MMP-2 (gelatinase A) and MMP-9 (gelatinase B) in the tissue extracts (Heussen and Dowdle, 1980; Hofmann *et al*, 1999). Each extract was diluted 1 : 1 with sample buffer (0.125 M Tris-HCl, pH 6.8, 17.4% (w v^−1^) glycerol, 4% sodium dodecylsulphate (SDS) and 0.01% bromophenol blue) and heated at 60°C for 20 min. After centrifugation for a few seconds 5 μl aliquots were loaded on a 7.5% (w v^−1^) standard Laemmli SDS-polyacrylamide gel containing 2 mg ml^−1^ gelatin (Type A: from Porcine Skin, Sigma) as a substrate. Gels were electrophoresed at 15 mA per gel while stacking and at 20 mA per gel during the separating phase until the bromophenol blue stained front reached the bottom of the gel. Collagenase from *Clostridium histolyticum* (Type VII, Sigma) was electrophoresed on each gel as an internal standard. After running, the gels were washed three times in 2.5% (v v^−1^) Triton X-100 for 10 min at room temperature to remove SDS. After washing twice in a buffer containing 50 mM Tris-HCl, 5 mM CaCl_2_ and 0,1% Triton X-100 (pH 7.8), the gels were incubated overnight at 37°C in the same buffer under gentle agitation. Zymograms were stained for 45 min with 0.25% (w v^−1^) Coomassie Brilliant Blue R250 dissolved in 40% methanol and 10% glacial acetic acid. Gels were destained twice for 10 min in the same solution without Coomassie Blue. Proteolytic activities were visualised by clear zones against a dark blue background indicating lysis of gelatin. Loading of the gels was such that proteinase activity was linear with the gelatin lysis. Quantification of the proteinase activities, which were expressed as arbitrary units per mg protein on the basis of size of the lysed area and intensity, was performed using a Sharp JX-330 scanner and Imagemaster ID software (Amersham Pharmacia Biotech, Uppsala, Sweden). In-between comparison of values, obtained on different gels, was performed using the internal standard that was present on each gel. The presence of true MMP activity was confirmed by adding 10 mM EDTA or 1 mM 1,10′ phenanthroline, both MMP inhibitors, to the buffers used after electrophoresis. Also human recombinant MMP-2 (1 ng per lane) and -9 (0.5 ng per lane; Oncogene Research Products, Cambridge, MA, USA) were added sometimes to compare their location with endogenous activities (e.g. Figure 1

). Activation of recombinant proform MMPs was achieved by incubation with 1 mM p-amino-phenylmercuric acetate (APMA).

### Fluorometric MMP activity assay

Broad-spectrum MMP activity was determined in 71 out of 73 CRC patient samples (in two cases insufficient sample was left after zymography to perform the activity assay) using the quenched fluorogenic peptide substrate TNO211-F (Dabcyl-Gaba-Pro-Gln-Gly-Leu-Cys(Fluorescein)-Ala-Lys-NH_2_) essentially as described previously (Beekman *et al*, 1999; DeGroot *et al*, 2001). The substrate is mainly cleaved by the gelatinases (MMP-2 and -9) and collagenase 3 (MMP-13) and to a lesser extent by collagenase 2 (MMP-8) and membrane-bound metalloproteinase (MT1–MMP). As such, conversion of this substrate mainly reflects gelatinolytic activity. In short, substrate hydrolysis by tissue extracts was determined in the presence of EDTA-free general proteinase inhibitor Complete (Roche Molecular Biochemicals, Indianapolis, IN, USA) to prevent conversion of TNO211-F by proteinases other than MMPs. Further improvement of the assay specificity for MMPs was achieved by determining the difference in substrate conversion in presence and absence of synthetic MMP inhibitor BB94 (10 μM). The rate of substrate conversion (RFU per s; relative fluorescence units per second) at 30°C (λ_ex_: 485 nm, λ_em_: 530 nm; Cytofluor4000, PerSeptive Biosystems) was normalised to the amount of protein present in the tissue extract and expressed as RFU per s per mg protein. All samples and reagents were diluted in buffer containing 50 mM Tris (pH 7.5), 5 mM CaCl_2_, 150 mM NaCl, 1 μM ZnCl_2_, 0.01% Brij-35 and 0.02% NaN_3_.

### Statistical analysis

Comparisons between MMP levels in matched tumour tissue, transitional tissue and distant normal mucosa were performed using the Friedman two-way ANOVA. As a post-test procedure for pairwise comparisons Dunn's post-test was used. The relationship between MMP levels and clinicopathological parameters was analysed using the Mann–Whitney *U*-test when comparing data between two groups; Kruskal–Wallis test was used when comparing more than two groups, with, in-between comparisons using Dunn's post-test. Differences were considered statistically significant at the *P*<0.05 level.

## RESULTS

### Proteinase activity by gelatin zymography

Seventy-three paired tissue samples of CRC tumour tissue and matching normal mucosa were extracted and analysed by quantitative gelatin zymography. In addition, gelatinase activity was also determined in transitional (tumour to normal) tissue which includes the invasive edge. Figure 1 shows a zymogram containing tissue extracts from three CRC patients, representative for all samples investigated. Clearly, the extracts contained multiple proteinase species of varying molecular weight exhibiting gelatinase activity. All were completely inhibited by EDTA (not shown) confirming their status as metalloproteinases. Comparison with the purified MMPs showed the presence of proMMP-9 (92 kDa), active MMP-9 (82 kDa), proMMP-2 (72 kDa) and active MMP-2 (62 kDa). The lytic bands of high molecular weight forms, found at approximately 220 and 135 kDa, closely correlated with both each other (*r*=0.96) and with those of proMMP-9 (*r*=0.97 and 0.96 for the 220 and 135 kDa bands respectively).

**Figure 2:**
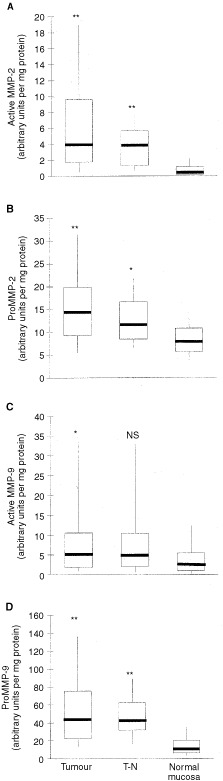
A comparison of MMP activity in CRC tumour (*n*=73), transitional tissue (T–N, *n*=33) and normal mucosa (*n*=73). Data are shown for active MMP-2 (**A**), proMMP-2 (**B**), active MMP-9 (**C**) and proMMP-9 (**D**). Horizontal bars represent the median values, boxes represent the interquartile range, vertical lines represent the 10 to 90% range of the observations. Enzyme activity, as measured by quantitative zymography, is expressed as specific activity in arbitrary units per milligram protein. Significant differences with normal mucosa: **P<0.001; *P<0.01.

All bands representing active and latent forms of MMP-2 and -9 were quantified and their gelatinolytic activity (on basis of protein) was calculated. There was a significantly higher level of active MMP-2, proMMP-2, active MMP-9 and proMMP-9 in tumour tissue compared to normal mucosa (*n*=73; *P*<0.001, *P*<0.001, *P*<0.01 and *P*<0.001 respectively) (Figure 2

). Active MMP-2, proMMP-2 and proMMP-9 were also higher in transitional tissue than in normal mucosa (*n*=33; *P*<0.001, *P*<0.01, *P*<0.001 respectively), but active MMP-9 was not. Median values in tumour and transitional tissue were essentially the same. Table 1

**Table 1:**
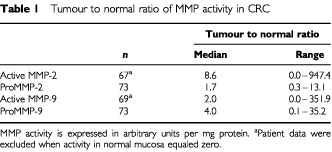
Tumour to normal ratio of MMP activity in CRC

shows the relative increase in MMP activity in tumour over normal tissue: the median tumour to normal ratio was highest (8.6) for active MMP-2 and lowest (1.7) for proMMP-2.

**Figure 3:**
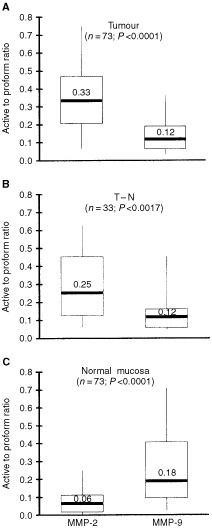
Ratio between active enzyme and proenzyme for both MMP-2 and -9. Data were obtained by quantitative gelatin zymography. (**A**) CRC tumour tissue; (**B**) transitional tissue (T–N); (**C**) normal mucosa. Horizontal bars represent median values, boxes represent the interquartile range, vertical lines represent the 10 to 90% range of the observations. *P* values represent the statistical differences between matched data on the MMP-2 and MMP-9 ratios.

Active MMP-2 and MMP-9 were not detectable in six and four samples from normal mucosa, respectively. In all other samples, levels of both pro- and active enzymes could be measured. The separate quantification of both the proenzyme and active enzyme also allowed the calculation of the ratio active/proenzyme in the three different tissues (Figure 3

). In normal mucosa, the ratio was three times higher (*P*<0.0001) for MMP-9 than for MMP-2. For MMP-2 this ratio increased five-fold (*P*<0.001) in tumour tissue and four-fold (*P*<0.001) in transitional tissue, as compared to normal mucosa. In contrast, for MMP-9 the ratio was highest in normal mucosa and decreased (*P*<0.05) in the samples containing tumour tissue.

### Correlation between MMP activity and clinicopathological parameters

**Figure 4:**
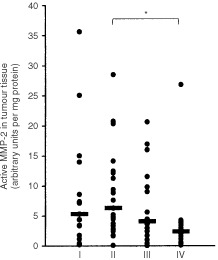
Active MMP-2 in primary CRC tissue extracts of stage I (*n*=15), II (*n*=23), III (*n*=16) and IV (*n*=17) tumours determined by quantitative gelatin zymography. Horizontal bars represent median values. Stage II versus stage IV: *P*<0.05.

The relations between clinicopathological parameters and the levels of active and proform MMP-2 and -9 in tumour tissue are shown in Table 2. A number of correlations was found for MMP-2 but only one for MMP-9. ProMMP-9 levels showed a significant correlation with tumour location (*P*=0.040). Rectum tumours showed the lowest levels of proMMP-9 whereas the left colon showed the highest levels. Neither age, tumour size, histological differentiation, tumour cell mucin, T stage nor lymph node involvement correlated with gelatinolytic MMP activity. In normal mucosa male patients displayed higher levels of proMMP-2 compared to female patients (*P*=0.011), but in tumour tissue no such difference was seen. Comparison between primary tumours with and without synchronous liver metastasis showed that tumours with liver metastasis (TxNxM1) had significantly lower activity of both active MMP-2 (*P*=0.005) and proMMP-2 (*P*=0.009) than those without liver metastasis (TxNxM0). These differences were also observed in distant normal mucosa, but statistical significance was only reached for the activated form of MMP-2 (*P*=0.011). The absolute activity of these MMP-2 levels in normal mucosa were, however, much lower (approximately 10 times) than those in tumour tissue. The active to proenzyme ratio of MMP-2 did also differ with this parameter. In both tumour and distant normal mucosa the ratio was significantly lower in the TxNxM1-group than in the TxNxM0-group (*P*=0.007, *P*=0.002 respectively). Comparing the MMP activity with stage of disease, a trend was observed towards lower levels of active MMP-2 and proMMP-2 with advanced stage of disease in tumour (*P*=0.024 and *P*=0.044 respectively) and in normal mucosa (*P*=0.019 and NS respectively). This also applied to the MMP-2 active to proenzyme ratio in tumour (*P*=0.028) and normal mucosa (*P*=0.002). In detail differences were most explicit between stage II (T3–4NxM0) and stage IV (TxNxM1) tumours for active MMP-2 (Figure 4

) and the MMP-2 active/proenzyme ratio (both *P*<0.05).

### Fluorometric MMP activity assay

**Figure 5:**
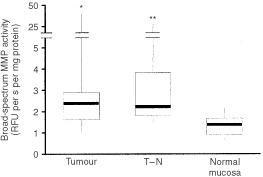
Broad-spectrum MMP activity in CRC tumour (*n*=71), transitional (T–N; *n*=29) and normal tissue (*n*=71) determined by quenched fluorogenic substrate hydrolysis. Activity is expressed as the rate of substrate conversion on basis of protein. Horizontal bars represent median values, boxes represent the interquartile range, vertical lines represent the 10 to 90% range of the observations. *P<0.01, **P<0.001 versus normal mucosa.

Nearly all samples were also measured with a broad-spectrum MMP assay, using a substrate, which is mainly recognised by the gelatinases. Figure 5

shows that overall gelatinolytic MMP activity was significantly higher in both tumour (*P*<0.01) and transitional (*P*<0.001) tissue than in normal mucosa. In contrast to the MMP-2 activities as determined by zymography, broad-spectrum MMP activity in tumour tissue did not correlate significantly with any of the clinicopathological parameters mentioned in Table 2. The only difference found, was that the transitional tissue of patients with distant metastatic liver disease (*n*=5) showed higher activity (3.9 versus 2.0, medians in arbitrary units, *P*=0.032) compared to patients without distant metastases (*n*=24).

## DISCUSSION

The present data show that tumour tissue contains higher levels of active and proform MMP-2 and -9 than distant normal mucosa in patients with CRC, with the increase in active MMP-2 being the most pronounced. The active to proform ratio of MMP-2 is highest in tumour tissue, whereas this ratio for MMP-9 is highest in distant normal mucosa. Furthermore, MMP-2 levels do inversely correlate with stage of disease.

The level of proteinase activity in this study was measured using gelatin zymography and broad-spectrum MMP substrate hydrolysis. Zymography separates the proforms and active forms of MMPs but the technique will not distinguish between free MMPs and those complexed with their natural inhibitors, the TIMPs (Kleiner and Stetler Stevenson, 1994). Zymography, therefore, yields no absolute values on the levels of active and latent MMPs *in vivo*, but rather a representation of the levels of the active and latent forms in both free and complexed form in the various tissues studied. The results should always be interpreted keeping this in mind. The broad-spectrum MMP activity assay, however, does not measure TIMP-complexed MMP activity. Since levels of TIMPs have not been determined in this study, one could speculate that the increased levels of MMP-2 and -9 in tumour will have no actual biological consequence in the *in vivo* situation because of endogenous inhibition.

However, in a recent study it is shown that the level of TIMP-2 protein, when measured with ELISA, is significantly lower in colorectal tumours than in normal mucosa (Baker *et al*, 2000). With the high levels of active MMP-2 present in tumour, total inhibition seems therefore unlikely. Moreover, using the MMP activity assay with a fluorogenic peptide substrate, enhanced MMP activity was observed in tumour tissue. Since this assay would not measure MMP activity if all MMPs were inhibited by TIMPs this indicated that indeed the increased MMP activation is not compensated for by increased TIMP production. Also, the ability of TIMP-1, which is reported to be upregulated (Baker *et al*, 2000), to bind with all MMP-9, can be disputed. TIMP-1 availability should prevent formation of MMP-9 dimers (Goldberg *et al*, 1992), while we found high levels of the homodimer of MMP-9 (220 kDa, Figure 1) and the heterodimer of MMP-9 and NGAL (135 kDa) (Zeng *et al*, 1999), indicating that there is unbound MMP-9 left in tumour tissue.

Gelatin zymography demonstrates that both active and proMMP-2 and -9 levels are elevated significantly in primary CRC tissue compared to normal mucosa. These observations are consistent with previous observations on active and proform MMP-2 and proMMP-9 (Liabakk *et al*, 1996). The presence of active MMP-9 has also been described before (Yamagata *et al*, 1991; Garbett *et al*, 1999a; Zeng *et al*, 1999; Baker *et al*, 2000), but quantitative measurement in all samples had not been reported up until now. Active MMP-2 increases more than its proform in tumour tissue. Thus there is not only more MMP-2 present in tumour than in normal tissue, but the ratio active to proenzyme also increases substantially. A similar finding was reported by Parsons *et al* (1998) and McKerrow *et al* (2000) and supports the view that the activation of MMP-2 is a crucial step in tumour invasion. Expression of MMP-2 mRNA in tumour has been described in stromal fibroblastic cells (Poulsom *et al*, 1992; Newell *et al*, 1994), but MMP-2 protein appears to be localised predominantly in the cytoplasm of tumour cells (Ring *et al*, 1997). A cell membrane-bound metalloproteinase (MT1-MMP), present on tumour cells, has been shown to activate proMMP-2 (Kikuchi *et al*, 2000) and is most likely responsible for anchoring MMP-2 to these malignant cells. Overexpression of MT1-MMP and enhanced MMP-2 protein activity may thus be induced in the process of tumour progression.

In contrast to MMP-2, the proform of MMP-9 increases much more than active MMP-9 during normal to tumour conversion. This finding is new since previous studies reported difficulty in measuring the active form, because of its unstability (Parsons *et al*, 1998) or presumed low levels (Liabakk *et al*, 1996). In CRC it has been shown that MMP-9 mRNA is expressed in macrophages especially in the invasive site. Immunoreactivity has been shown in macrophages and neutrophils but not in tumour cells (Nielsen *et al*, 1996; Zeng and Guillem, 1996). It is suggested that MMP-9 levels reflect the presence of inflammatory infiltrate around the tumour rather than the characteristics of the tumour itself. However, Parsons *et al* (1998), did not find a correlation between MMP-9 and the amount of peritumoural lymphocytic infiltrates. Our observation that proMMP-9 increases more than its active form in tumour tissue may result from induced secretion of large amounts of proform by macrophages and neutrophils. As yet, we cannot explain why the activation of the proMMP-9 form to the active form, as represented by the active to proform ratios, appears to be lower in the tumour than in normal tissue. However, it would be unjustified to conclude from this that the role of MMP-9 in tumour invasion and progression would be less important than that of MMP-2, which shows increased activation in tumour. MMP-9 may even be a more effective protease than MMP-2 with respect to substrates in the extracellular matrix, as it is *in vitro* for gelatin during zymography. Still, it remains to be established if MMP-9 plays a significant role in tumour progression in CRC. It is evident, however, that there are significant differences in the state of activation between the gelatinases, which might be indicative for their different roles in the progression of cancer.

Comparison between clinicopathological parameters and gelatinase activity reveals correlations for MMP-2, in particular in relation to stage of disease. Neither Liabakk *et al* (1996) nor Parsons *et al* (1998) found such a correlation, but these authors have not included stage IV tumours in their studies. Indeed, in our series activities are found in stage I, II and III tumours similar to those of Parsons *et al* (1998), but a significantly lower activity of both active and proMMP-2 is found in metastatic stage IV tumours if compared to all tumours without distant (c.q. liver) metastases. It has been suggested that MMP activities in the separate disease stages may vary because of differences in stromal components surrounding the tumours (Liabakk *et al*, 1996). Although this seems reasonable in view of the fact that different cell–cell contacts and specific cell–matrix interactions may cause changes in MMP expression (DeClerck, 2000), one would then also expect variations in MMP activity with depth of invasion. However, we find no correlations between MMP activity and T stage. The finding that active MMP-2 in normal mucosa is also inversely correlated with stage of disease and with distant metastatic disease is remarkable. Although the activities of active MMP-2 were much lower, they show a similar trend. This may be due to the effect of local diffusion or perfusion via lymph or blood vessels. Previous studies on MMP activities have not compared normal tissue MMP activities with clinicopathological parameters, most probably due to difficulties measuring these low activity levels.

The observation that in normal mucosa the level of proMMP-2 is higher in males than in females has not been described before. Its relevance in cancer is doubtful because these differences are seen in normal tissue only, where activities are very low, and do not pertain to active MMP-2. Also, the correlations between proMMP-2 levels and other clinicopathological parameters do not result from a disbalance in gender.

The correlation between tumour proMMP-9 levels and colonic location has not been found before. Zeng *et al* (1996) did not find correlation between MMP-9 mRNA expression and location. This probably means that the increased presence of the proenzyme is caused by upregulation at the translational level. While no further significant correlations between MMP-9 activity and clinicopathological parameters in either tumour or normal tissue were found, a trend towards lower levels of MMP-9 with advancing stage seems apparent (Table 2). Using immunohistochemistry such a trend has also been observed by Takeha *et al* (1997), who suggest that MMP-9 is, next to being a tumour-promoting factor, also related to the host defence mechanism. The host reaction against the tumour counteracts hematogenous metastasis and local invasive growth. If so, early or slowly invading and growing tumours may trigger more explicit effects of this host defence mechanism, in this case production of MMP-9, than fast and diffuse invading tumours.

We have also performed assays on extracts from transitional tissue. These samples contain, next to tumour and adjacent normal tissue, the invasive edge. Since only 33 matched samples were available, analysis of relations between MMP activity and clinicopathological parameters is not feasible. Transitional tissue and tumour tissue contain similar levels of active MMP-2, -9 and broad-spectrum MMP activity, with the first and latter being significantly higher than those in distant normal mucosa. One could speculate that proteinase activity is higher in the invasive edge than in the tumour centre and that overall activity in the extract from transitional tissue is lowered by the presence of the normal tissue. However, it has been reported that the adjacent ‘normal’ mucosa also exhibits increased levels of active MMP-2 and -9 (Yamagata *et al*, 1991; Poulsom *et al*, 1992). Thus, any definite conclusions regarding proteinase activity in the invasive edge must await further analysis by means of, for instance, microdissection or *in situ* zymography.

Broad-spectrum MMP activity was measured with a fluorogenic substrate assay. As with zymography, increased activity is present in both tumour and transitional tissue. A variety of MMPs may be responsible for this observation since the substrate is hydrolysed by all MMPs that exhibit gelatinolytic activity (e.g. MMP-2, -9 and -13). By the use of a specific MMP inhibitor as a control in this activity assay and the use of a general protease inhibitor, only actual active MMPs are measured and not other proteinases or protein-inhibitor complexes. This is an important difference with assays used by others (Garbett *et al*, 1999b; Baker *et al*, 2000; McKerrow *et al*, 2000). The results obtained here indicate that in the tumours studied MMP (production and) activation is elevated compared to the production of TIMPs. This results in an excess of free, active MMPs, which may contribute to the invasiveness of the tumour.

In summary, our results show increased levels of MMP activity in tumour and transitional tissues compared to normal mucosa in CRC patients. Explicit differences between MMP-2 and MMP-9, with respect to both levels of active enzyme and correlations with clinicopathological parameters, strongly suggest different roles for the gelatinases in tumour progression. The results emphasise the necessity to collect data on the proteinase activity of individual MMPs in order to develop suitable intervention strategies.
